# An Unusual Presentation of Takotsubo Syndrome in a Young Female of Middle Eastern Origin

**DOI:** 10.1002/ccr3.70166

**Published:** 2025-03-06

**Authors:** Deepak Lal, Safaa AlMohdar, Mohammed Elsayed Tawfik Elsayed, Humariya Heena

**Affiliations:** ^1^ Department of Cardiology Zayed Military Hospital Abu Dhabi UAE; ^2^ Medical Education Department Zayed Military Hospital Abu Dhabi UAE

**Keywords:** acute coronary syndrome, acute ischemic events, stress‐induced cardiomyopathy, Takotsubo syndrome, ventricular wall motion abnormality

## Abstract

This case of Takotsubo syndrome in a young, non‐stressed female highlight that it can present across various ages and ECG patterns, suggesting a need for further genetic research. In ECGs showing ST‐elevation myocardial infarction (STEMI), non‐invasive coronary evaluation should be prioritized over direct angiography. Echocardiographic GLS strain can help predict prognosis and facilitate early recovery.

## 1 Introduction

Takotsubo Syndrome, commonly known as stress‐induced cardiomyopathy, has been reported as an intriguing topic of discussion in many new studies [1]. Japanese authors initially coined the name, which refers to the characteristic shape assumed by the left ventricle towards the end of systole, similar to octopus traps in Japan. A noticeably increasing trend has been noted in the cases presented in the past 5 years, reported from Europe, America, and Australia [2, 3].

This pathology is estimated to account for 1%–2% of all patients coming into clinical attention for acute ischemic events. Recent statistics reported by the American Heart Association show that out of the staggering 732,000 yearly dismissals with a primary diagnosis of acute MI, 7000–14,000 may be attributable to stress‐induced cardiomyopathy [4].

A literature search on PubMed revealed a significant number of cases demonstrating left ventricular ballooning at atypical sites, including the median ventricular level, the base of the ventricle, the inferior wall, or the anterior wall.

The Mayo Clinic has devised a diagnostic criterion comprising four key points, and fulfilling all four conditions is required for an assertive diagnosis of this disease. The conditions are given below: [1, 5].

1. Transitory decline in LV function that restores in time.
2. Exclusion of CAD and angiographic evidence against acute plaque rupture.
3. Newly developed ECG abnormalities and/or a significant elevation in cardiac enzymes.
4. Absence of any other pathology, such as pheochromocytoma or myocarditis.

In a first‐of‐its‐kind report from the Middle East, we report a case of Takotsubo cardiomyopathy (TCM) with ventricular ballooning at the inferior wall in a patient who presented with chest pain and ECG findings suggestive of ischemia.

## 2 Case History

A 28‐year‐old female with no known co‐morbidities presented to the Emergency Department of a tertiary care hospital in the early morning hours with complaints of chest pain and palpitations. As revealed on further interrogation, the pain started in the chest, was crushing in character, radiating towards the shoulder, arm, and forearm, and was gradually worsening. It was associated with mild sweating and palpitations and was not relieved by local Non‐Steroidal Anti Inflammatory Drugs (NSAIDs). There was no prior history of such episodes and no history of active or passive smoking, drug abuse, depression, stress triggers, dyslipidemias, or family history of Coronary Artery Disease (CAD). The vitals at the time of presentation were as follows: Blood pressure: 100/70, Heart rate: 100 beats/min, Temperature: Afebrile, Respiratory rate: 17 breaths/min.

## 3 Differential Diagnosis, Investigations, and Treatment

**FIGURE 1:**
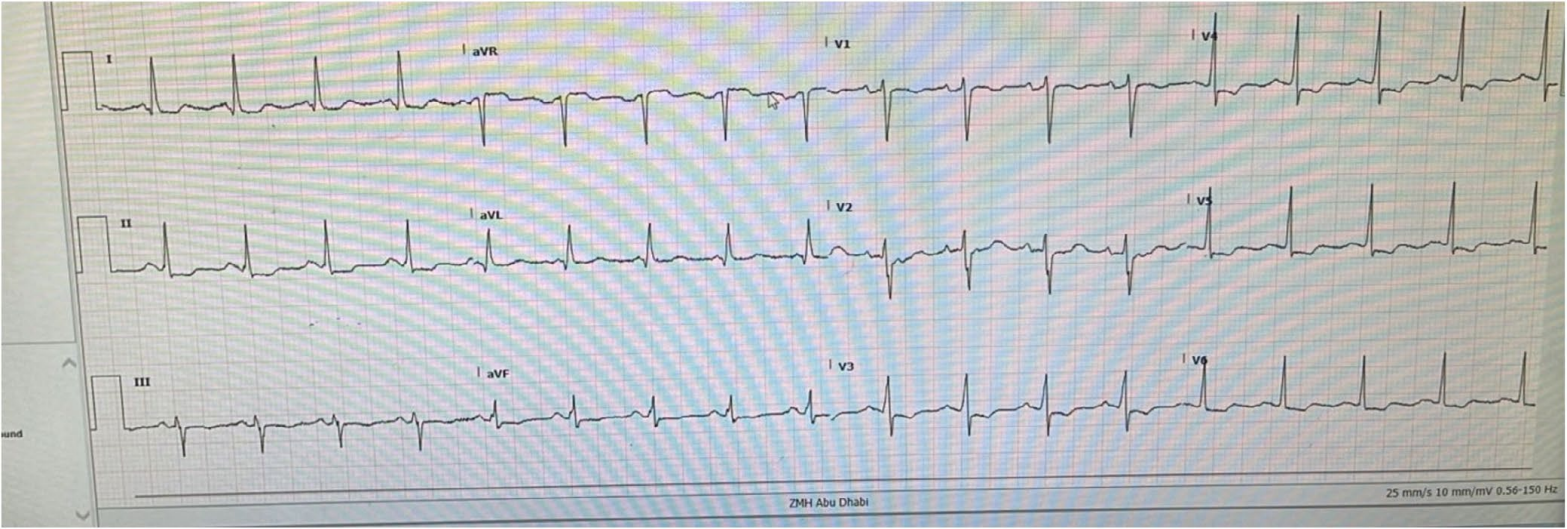
Electrocardiogram which shows global ST‐segment depressions, most prominent in Leads II, aVF, and V2‐V6, along with T‐wave inversions in V3‐V6.

A presenting Electrocardiogram (ECG) was obtained (Figure 1), which showed global ST‐segment depressions, most prominent in Leads II, aVF, and V2‐V6, along with T‐wave inversions in V3‐V6. High‐sensitivity Troponin levels were recorded as 1.639 ng/dL (normal < 0.063 ng/dL). The patient was immediately treated per the Acute Coronary Syndrome (ACS) protocol under the impression of an ischemic attack.

**FIGURE 2:**
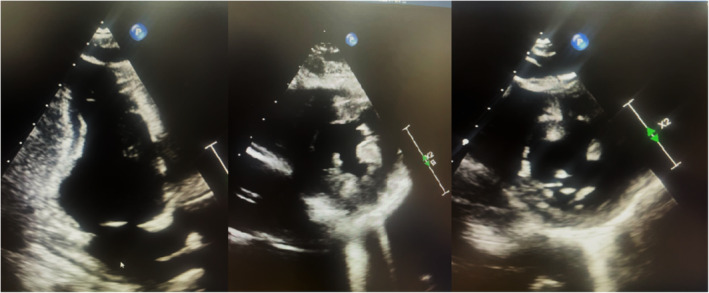
An echocardiograph of the patient shows inferior and basal segment hypokinesia and an Ejection Fraction of 50%.

**FIGURE 3:**
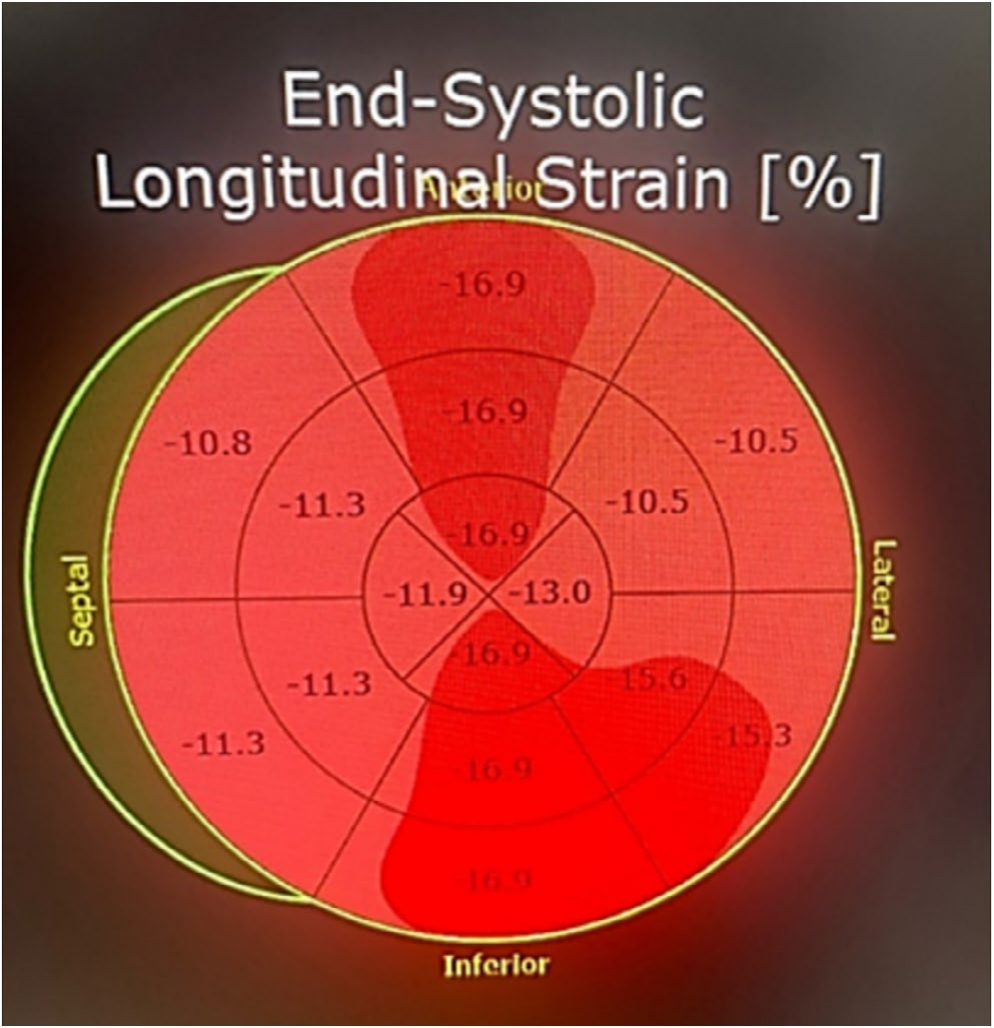
GLS score of −14 at presentation.

**FIGURE 4:**
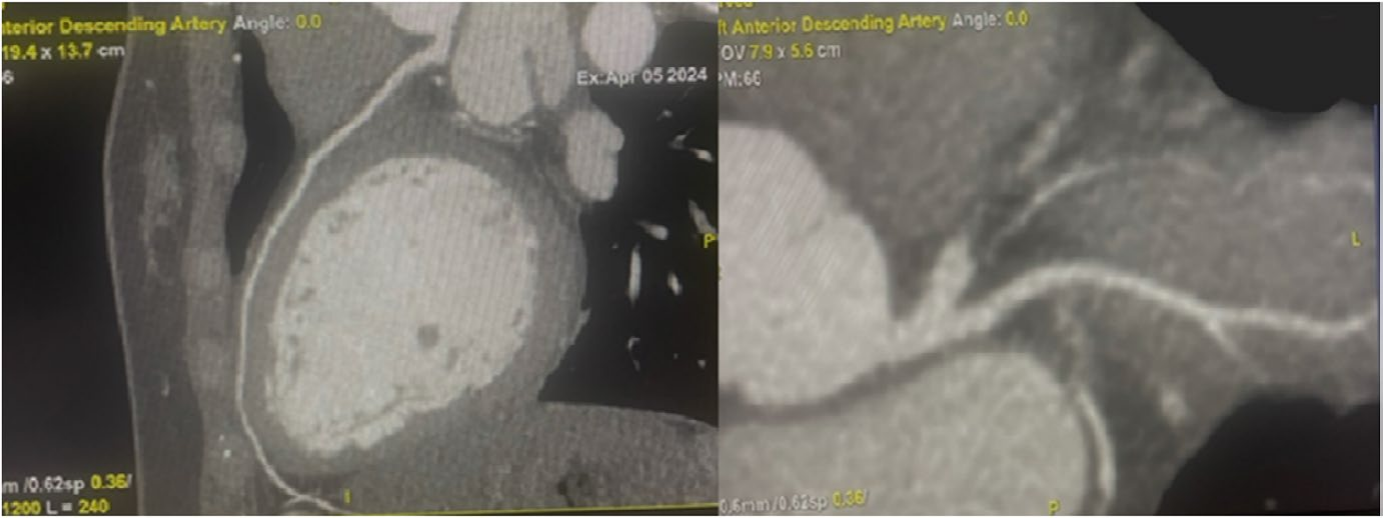
CT angiography of the patient showing unremarkable anatomical variation and nonobstructive coronaries.

After the initial stabilization of the patient, Echocardiography was done, which elucidated inferior and basal segment hypokinesia and an Ejection Fraction of 50% (Figure 2), with a Global Longitudinal Score (GLS) of −14 (Figure 3). The typical presentation of ACS, yet the unremarkable history and unsupportive biodata of the patient presented a diagnostic dilemma for the physicians, who suspected a congenital anomalous coronary artery disease which might have been responsible for the development of ischemia in a young female. A cardiac CT angiography yielded an unremarkable anatomical variation in the patient and also excluded any obstructive lesions (Figure 4).

**FIGURE 5:**
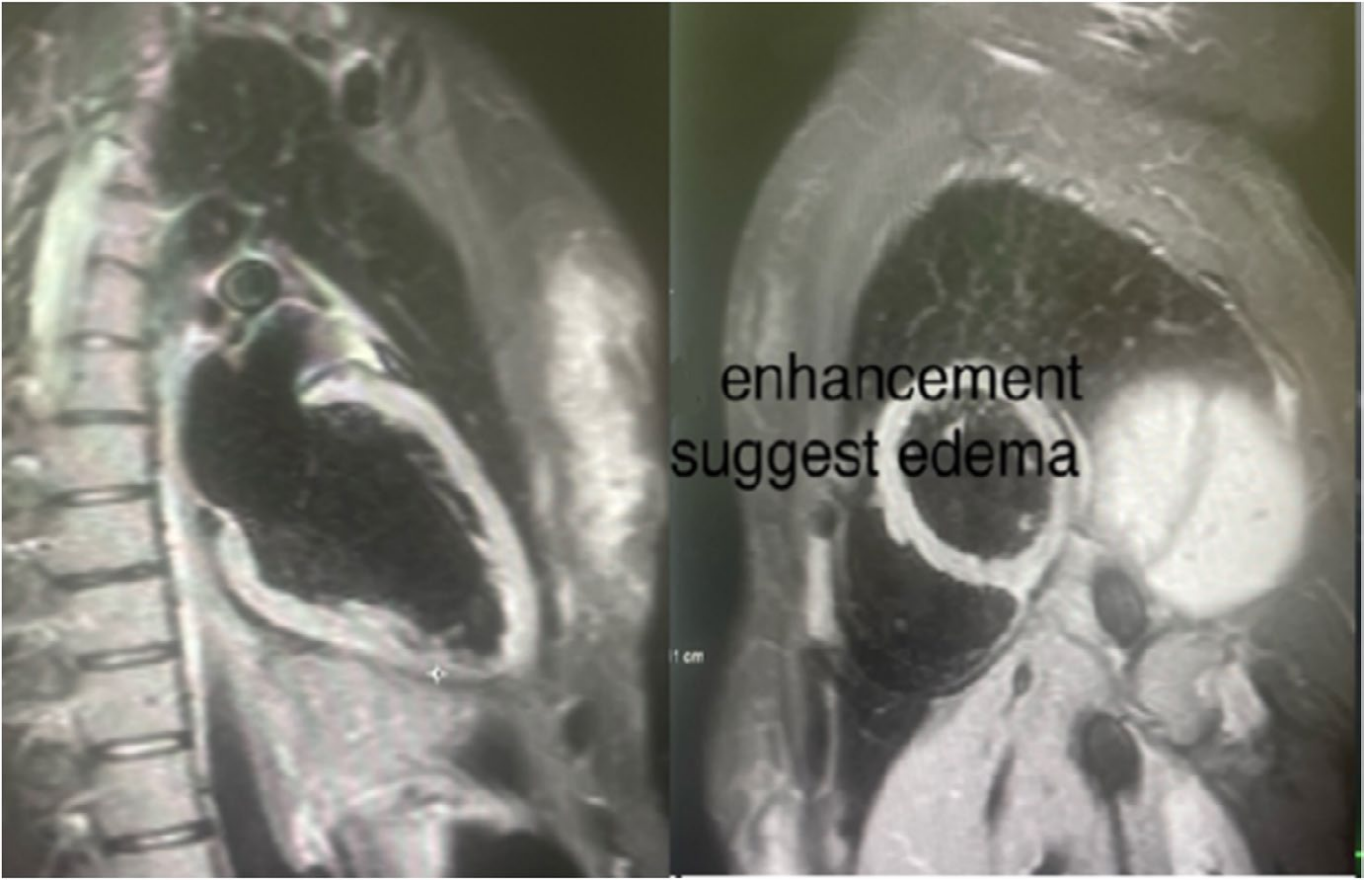
Cardiac MRI showing normal Late Gadolinium Enhancement and mild edema at the inferior wall segment.

Next, a cardiac MRI reiterated the echo findings, with normal Late Gadolinium Enhancement (LGE) and mild edema noted at the inferior wall segment. Also, there was no lesion present (Figure 5). This pointed towards a transient wall motion abnormality with segmental involvement. We ruled out coronary artery disease and specific myocarditis. She was managed as ACS with dual antiplatelet therapy and b blocker bisoprolol 2.5 mg per oral. The patient was observed in the Cardiac Care Unit for a day, after which she was stepped down as her symptoms improved. Subsequently, the diagnosis of ischemic attack was excluded, anticoagulants were withdrawn, and she was put on Bisoprolol 2.5 mg OD during the rest of her hospital course. Her sequential Troponin levels showed a steady decline towards the therapeutic range, from 0.211 ng/dL to 0.048 ng/dL. During the hospital stay of 3 days, she was completely symptom‐free and was discharged on tablet Bisoprolol 2.5 mg OD.

## 4 Outcome and Follow‐Up

**FIGURE 6:**
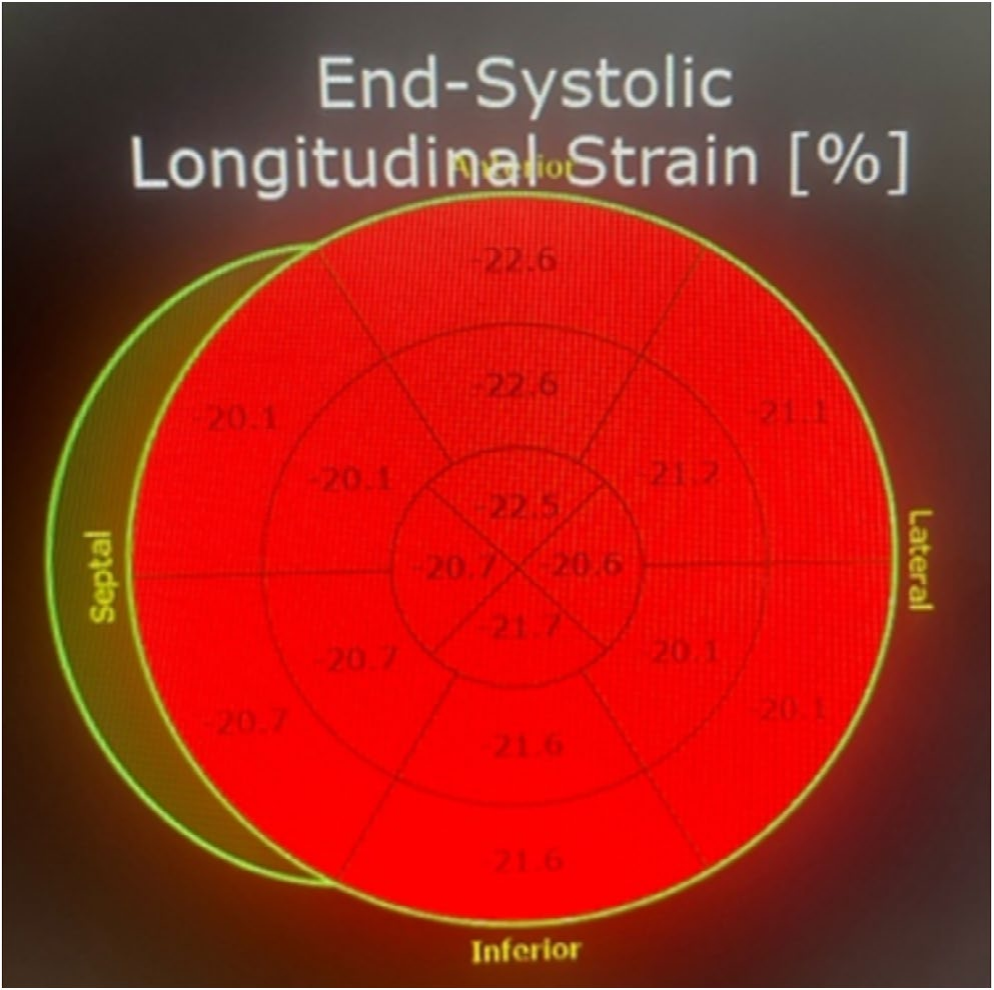
GLS score of −21 on follow‐up.

She was followed up in the OPD after 4 weeks, where a repeat work‐up revealed a normal Left Ventricular wall motion and a normally contracting inferior wall. Her GLS also improved to −21 from −14 (Figure 6). The complete resolution of her cardiac function was a testament to the diagnosis of Takotsubo Syndrome with focal involvement and non‐specific ECG changes.

## 5 Discussion

TCM is a rare clinical diagnosis with a prevalence estimated between 0.7%–2.5% of patients presenting with troponin‐positive suspected ACS [6]. Although the obscure etiology of TCM is still unclear, the clinical ubiquity of the disease has seen a rise as more cases are reported [7]. Postmenopausal women within the range of 58–75 years old are a preeminent risk factor for this condition, with 90% of cases presenting within this age group [7]. However, our case was a young female with no history of stress with NSTEMI which is uncommon in TS. Despite the meager amount of literature available to establish prominent triggers and pathophysiology, there are several plausible factors described in the International Takotsubo Registry (ITR), based on 1750 patients. A physical trigger contributed to 36% of cases, while 28.5% of patients had no triggers at all. 27.7% had emotional liabilities, and 7.8% of patients had both emotional and physical stressors [8, 9].

Among the variants of TCM, the classical type (also known as apical type; apical akinesia, basal hyperkinesia) is the most common variant (82%), followed by the midventricular type (14.6%), the reverse type (2%, also known as basal type; basal akinesia, apical hyperkinesia), and the focal type (1.5%) [9, 10, 11]. Herein we had a focal involvement of the inferior segment with normal EF and low GLS.

Wall motion abnormalities must be appreciated via imaging and all potential causes of these abnormalities are elaborated to establish a diagnosis of TCM. The advent and addition of Magnetic Resonance Imaging (MRI) into cardiovascular imaging (CMRI) has proved to be a milestone when high‐quality imaging is required. The results have been promising when applied to exempt other entities, such as myocarditis or myocardial infarction (MI), and validate the diagnosis of TCM [12]. The myocardium is noticeably inflamed during the acute phase of TCM and is visualized as edema on CMRI, where the edematous region corresponds to the pathological area of wall motion abnormality. However, this can lead to a false‐positive diagnosis as the findings are not exclusive to TCM and may also be observed in myocarditis or MI. This warrants the usage of gadolinium‐based contrast, which is adopted by a technique known as late gadolinium enhancement (LGE), which can discriminate between TCM and other diagnoses, as the presence of LGE is testimonial in MI or myocarditis but is usually absent in TCM [12, 13].

The reversible, transient left ventricular systolic function so particular to TCM is also described in cases of pheochromocytoma, acute brain injury as seen in subarachnoid hemorrhage, cerebrovascular events, and in various other neurologic conditions such a neurogenic stunned myocardium, where stress causing a catecholamine efflux is ordained to be the most plausible mechanism responsible. The histopathological picture, which is the manifestation of an upheaval of intracellular Ca^+2^ secondary to catecholamine surge, is distinguished by the finding of a contraction band necrosis, which is a shrunken area, walled‐off with hypercontracted sarcomeres, housing islands of lysed myocytes in a sea of mononuclear inflammatory cells and densely eosinophilic transverse bands [14].

TCM, due to drug administration, is a very interesting etiological factor that further consolidates the role of catecholamines in developing this benign condition. A systematic review of case reports published by Kido et al. navigates the 157 cases reported of drug‐induced TCM and elucidates the pragmatic relation between catecholamines and TCM, as 68.2% of these cases were the consequence of catecholamine‐related rugs. 11% of cases were reportedly due to coronary spasms, while 14.3% of cases did not have any identifiable cause, as in the case we have presented above [15].

TCM and ACS are often mistakenly misdiagnosed owing to the overlapping clinical presentation. As the primary investigations, it is an intriguing ordeal if ECG and Echo can discriminate between ACS and TCM, but regrettably, there is a lack of data establishing any compelling link between a unique ECG pattern or specific findings on echocardiography and TCM. Patients with TCM may present with ECG findings strongly suggestive of MI, and inconclusive echo findings with focal or diffuse involvement of the myocardial wall, posing a challenge in the differentiation based on these two modalities. A comparative study found that 56% of cases of TCM report ST segment elevations in the anterior leads, and no ST segment elevations in the remaining 44%, among which 17% are non‐specific or normal ECGs, while 17% reveal diffuse T‐wave inversions, and 10% show healed anterior infarctions [16].

There are currently no clinical guidelines about the absolute treatment regimen for patients with TCM; however, the standard heart failure medications comprising Beta‐blockers, ACE inhibitors, and diuretics are also employed in these cases. Patients are also counseled to avoid any identified trigger factors and are usually managed on Beta‐blockers to prevent further episodes. The case was managed only with b blockers because the EF was normal. In conclusion, we described and explained a rare case of TCM involving a focal segment in an atypical age group with a presentation mimicking a Non‐ST segment elevation Myocardial Infarction but with no identifiable triggers suggestive of aggravating TCM. Further genetic studies may explain the unusual presentation of our case and provide insights into a better health outlook for such patients.

## Author Contributions

**Deepak Lal:** conceptualization, data curation, investigation, writing – original draft. **Safaa AlMohdar:** data curation, supervision, writing – review and editing. **Mohammed Elsayed Tawfik Elsayed:** conceptualization, data curation, formal analysis, validation, writing – review and editing. **Humariya Heena:** conceptualization, data curation, investigation, writing – original draft.

## Consent

Written informed consent was obtained from the patient to publish this report in accordance with the journal's patient consent policy.

## Conflicts of Interest

The authors declare no conflicts of interest.

## Data Availability

Data available on request due to privacy/ethical restrictions.
